# Cortisol and C-Reactive Protein Vary During Sleep Loss and Recovery but Are Not Markers of Neurobehavioral Resilience

**DOI:** 10.3389/fphys.2021.782860

**Published:** 2021-11-29

**Authors:** Erika M. Yamazaki, Caroline A. Antler, Courtney E. Casale, Laura E. MacMullen, Adrian J. Ecker, Namni Goel

**Affiliations:** ^1^Biological Rhythms Research Laboratory, Department of Psychiatry and Behavioral Sciences, Rush University Medical Center, Chicago, IL, United States; ^2^Division of Sleep and Chronobiology, Department of Psychiatry, Perelman School of Medicine, University of Pennsylvania, Philadelphia, PA, United States

**Keywords:** cortisol, C-reactive protein, sleep deprivation, psychological stress, biomarkers, neurobehavioral performance, psychomotor vigilance test, karolinska sleepiness scale

## Abstract

Cortisol and C-reactive protein (CRP) typically change during total sleep deprivation (TSD) and psychological stress; however, it remains unknown whether these biological markers can differentiate robust individual differences in neurobehavioral performance and self-rated sleepiness resulting from these stressors. Additionally, little is known about cortisol and CRP recovery after TSD. In our study, 32 healthy adults (ages 27–53; mean ± SD, 35.1 ± 7.1 years; 14 females) participated in a highly controlled 5-day experiment in the Human Exploration Research Analog (HERA), a high-fidelity National Aeronautics and Space Administration (NASA) space analog isolation facility, consisting of two baseline nights, 39 h TSD, and two recovery nights. Psychological stress was induced by a modified Trier Social Stress Test (TSST) on the afternoon of TSD. Salivary cortisol and plasma CRP were obtained at six time points, before (pre-study), during [baseline, the morning of TSD (TSD AM), the afternoon of TSD (TSD PM), and recovery], and after (post-study) the experiment. A neurobehavioral test battery, including measures of behavioral attention and cognitive throughput, and a self-report measure of sleepiness, was administered 11 times. Resilient and vulnerable groups were defined by a median split on the average TSD performance or sleepiness score. Low and high pre-study cortisol and CRP were defined by a median split on respective values at pre-study. Cortisol and CRP both changed significantly across the study, with cortisol, but not CRP, increasing during TSD. During recovery, cortisol levels did not return to pre-TSD levels, whereas CRP levels did not differ from baseline. When sex was added as a between-subject factor, the time × sex interaction was significant for cortisol. Resilient and vulnerable groups did not differ in cortisol and CRP, and low and high pre-study cortisol/CRP groups did not differ on performance tasks or self-reported sleepiness. Thus, both cortisol and CRP reliably changed in a normal, healthy population as a result of sleep loss; however, cortisol and CRP were not markers of neurobehavioral resilience to TSD and stress in this study.

## Introduction

Chronic sleep deprivation is an important public health concern associated with many adverse health outcomes and clinical disorders such as anxiety, depression, immune dysfunction, Alzheimer’s disease, cardiovascular disease, obesity, cancer, and overall morbidity and mortality (Ferrie et al., 2007; Gallicchio and Kalesan, 2009; Mullington et al., 2009; Phan and Malkani, 2019; Al-Rashed et al., 2021). Sleep deprivation is a potent stressor generally resulting in marked increases in physiological sleepiness (e.g., the Maintenance of Wakefulness Test and the Multiple Sleep Latency Test) and subjective sleepiness [e.g., the Karolinska Sleepiness Scale (KSS)] and significant deficits in cognitive performance (Banks and Dinges, 2007; Goel et al., 2009; Casale et al., 2021a,b; Yamazaki et al., 2021a,b). However, a number of studies show there are robust and highly replicable phenotypic individual differences in response to repeated exposure to sleep deprivation: some individuals are resilient, and others are vulnerable to total sleep deprivation (TSD) and chronic sleep restriction (SR), both commonly experienced types of sleep loss (Van Dongen et al., 2004; Goel et al., 2009; Dennis et al., 2017; Yamazaki and Goel, 2020). Moreover, these inter-individual differences persist across months and years when exposed to repeated sleep loss (Dennis et al., 2017), and do not differ within various key subgroups, such as sex, age, race, and body mass index (Yamazaki and Goel, 2020).

Although well investigated, the effects of sleep deprivation on cortisol, a hypothalamic–pituitary–adrenal (HPA) axis marker, and C-reactive protein (CRP), an inflammatory marker, remain inconsistent: some studies report no change in cortisol (Vgontzas et al., 2004; Frey et al., 2007; van Leeuwen et al., 2009; Pejovic et al., 2013; Honma et al., 2020) or CRP (Faraut et al., 2011; Irwin et al., 2016; Choshen-Hillel et al., 2021), while others report decreases in cortisol (Åkerstedt et al., 1980) or CRP (Frey et al., 2007; Baek et al., 2020), or increases in cortisol (Leproult et al., 1997; Wright et al., 2015; Baek et al., 2020; Choshen-Hillel et al., 2021; Lamon et al., 2021) or CRP (Meier-Ewert et al., 2004; van Leeuwen et al., 2009). Similarly, studies have found that both acute and prolonged stress increase cortisol (Jönsson et al., 2010; Allen et al., 2014) and CRP (Eraly et al., 2014; Kennedy et al., 2014), although other studies reported no change in CRP (La Fratta et al., 2018; Szabo et al., 2020).

The Trier Social Stress Test (TSST) (Kirschbaum et al., 1993), a well-validated experimental acute psychological stressor, has been shown to increase cortisol (Jönsson et al., 2010; Kennedy et al., 2014; Allen et al., 2017) and CRP (Campisi et al., 2012; Kennedy et al., 2014) in healthy individuals. Notably, cortisol has shown varied responses to the combination of sleep loss and the TSST in the few studies that have investigated this combination; cortisol was blunted (Vargas and Lopez-Duran, 2017), increased (Minkel et al., 2014), or not significantly different (Schwarz et al., 2018) in those who experienced TSD and the TSST compared to those who experienced the TSST alone. To our knowledge, the CRP response to the combination of sleep loss and TSST has not yet been investigated.

Cortisol and CRP are associated with responses to stress (Eraly et al., 2014; Henckens et al., 2016), cardiovascular disease risk (Li et al., 2017; Crawford et al., 2019; Iob and Steptoe, 2019), and performance on multiple cognitive dimensions in dementia (Wersching et al., 2010; Hajjar et al., 2018; Ouanes et al., 2020). Moreover, low salivary cortisol is generally recognized as a marker for Posttraumatic Stress Disorder (PTSD) (Pan et al., 2018). However, to our knowledge, neither cortisol or CRP have been investigated as biomarkers for individual differences in cognitive performance or self-reported sleepiness responses to sleep loss or the combination of sleep loss and psychological stress.

Given the relationships of sleep deprivation and psychological stress with cortisol and CRP, these are novel, uninvestigated, candidate biomarkers that may identify individuals who are resilient or vulnerable to the combination of these stressors. We evaluated whether sleep loss, psychological stress and recovery affect cortisol and CRP levels and whether these biological markers could discern resilient and vulnerable individuals before and in response to TSD and psychological stress, which would have particularly important implications in applied settings, where both are commonly experienced (Barger et al., 2014; Cromwell et al., 2021). We hypothesized the following: (1) cognitive performance and self-rated sleepiness would be adversely impacted during TSD and psychological stress; (2) cortisol and CRP levels would increase during TSD and psychological stress and decrease with recovery; (3) resilient and vulnerable individuals (defined by each cognitive performance measure or by self-rated sleepiness) would show differential patterns of change in cortisol and CRP levels across the study; and (4) pre-study cortisol and pre-study CRP levels would distinguish cognitive performance and self-rated sleepiness during subsequent TSD and psychological stress.

## Materials and Methods

### Participants

We studied 32 healthy adults (ages 27–53; mean age ± SD, 35.1 ± 7.1 years, 14 females) in the Human Research Program Human Exploration Research Analog (HERA), a high-fidelity National Aeronautics and Space Administration (NASA) space analog isolation facility in Johnson Space Center in Houston, TX, United States. Groups of four participants at a time participated in one of the four 14-day studies or one of the four 30-day studies. Participants were thoroughly screened by NASA to ensure they had astronaut-like characteristics, including suitable educational or military experience (Nasrini et al., 2020; Smith et al., 2021). Participants were required to pass a drug screen and a physical exam, including an eye exam, ensuring they were in good health with no history of cardiovascular, neurological, gastrointestinal, or musculoskeletal problems, and underwent psychological assessment (Moreno-Villanueva et al., 2018; Nasrini et al., 2020; Smith et al., 2021). The study was approved by the Institutional Review Boards of NASA and of the University of Pennsylvania, and all protocol methods were carried out in accordance with approved guidelines and regulations. Participants provided written informed consent in accordance with the Declaration of Helsinki and received compensation for their participation.

### Procedures

**FIGURE 1:**
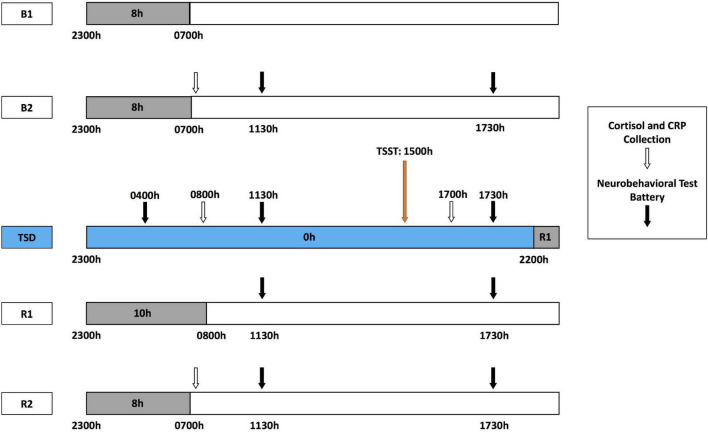
Five-day experimental protocol. The experimental protocol consisted of 2 days of baseline with 8-h time in bed (TIB) sleep opportunity (B1, B2; 2300–0700 h). Baseline cortisol and CRP collection (white arrows) occurred at 0700 h after the B2 sleep opportunity, followed by the neurobehavioral test battery (NTB) at 1130 and 1730 h (black arrows). After the B2 day, participants began 39 h of total sleep deprivation (TSD, blue block). During TSD, NTB administration occurred at 0400 h, cortisol and CRP collection at 0800 h, and NTB administration at 1130 h. A modified Trier Social Stress Test (TSST, orange arrow) was administered starting at 1500 h during the TSD day, with cortisol and CRP collection at 1700 h and NTB administration after TSST completion at 1730 h. Recovery followed TSD, including 10 and 8 h TIB sleep opportunities (R1 and R2, respectively). The NTB was administered at 1130 and 1730 h during R1 and R2, and cortisol and CRP collection occurred at 0700 h of R2.

During each HERA study, participants engaged in pre-study data collection, a 5-day experiment designed to induce sleep deprivation and psychological stress (Figure 1), and post-study data collection. The 5-day experiment consisted of 2 baseline nights [B1 and B2; 8-h time-in-bed (TIB), 2300–0700 h], followed by 39-h acute TSD (during which participants remained awake) that included a modified TSST conducted between 1500 and 1730 h after the TSD night to induce psychological stress (described below). TSD was followed by a 10-h TIB night of recovery (R1; 2200–0800 h), and a second 8-h TIB night of recovery (R2; 2300–0700 h). Fitness levels were not explicitly measured; however, all participants were in comparable good health, endured similar amounts of limited activity during the study, and were confined to engaging in prescribed activities at specific times. Napping was prohibited during the experiment. Sleep-wake episodes were verified objectively by wrist actigraphy (Philips Respironics Healthcare, Bend, OR, United States). Actigraphic sleep data were analyzed as in our prior studies (Dennis et al., 2017; Moreno-Villanueva et al., 2018; Yamazaki and Goel, 2020; Brieva et al., 2021; Casale et al., 2021b; Yamazaki et al., 2021a).

### Biomarker Collection

Salivary cortisol and CRP were collected at the following six time points: pre-study, B2, the morning of TSD (TSD AM), the afternoon of TSD (TSD PM), R2, and post-study (Figure 1). All collections were completed at the same time each day (0800 h before eating), except for the TSD post-stress assessment, which was collected at 1730 h. Pre- and post-study collections occurred 1 day before and 4 or 5 days after the study, respectively, in the same location as those collections during the 5-day experiment. All participants fasted for 10 h prior to all five AM collections and for 5 h prior to the one PM collection for consistency across the study and among participants.

#### Salivary Cortisol Collection and Processing

At each biomarker collection point, 1 mL of saliva was collected using Salivettes (Sarstedt, NC, United States). Following collection, these tubes were kept on ice until storage at −80°C for assay. Salivary cortisol levels were measured in duplicate using the Salimetrics cortisol ELISA kit (Salimetrics, PA, United States). Intra-assay and inter-assay coefficients of variation were 2.37 and 3.95%, respectively, and the minimum detectable value was 0.012 μg/dL. All samples from the same participant were measured in the same assay.

#### High-Sensitivity C-Reactive Protein Collection and Processing

At each biomarker collection time point, 4 mL of whole blood was collected in pre-cooled vacutainer tubes containing sodium heparin (BD, NJ, United States) and kept on ice until centrifugation at 4°C. After centrifugation, samples were immediately frozen at −80°C and stored until assay. Determination of high-sensitivity CRP concentrations were performed using an Immulite 1000 High Sensitivity CRP kit (Siemens Healthineers, PA, United States). Intra-assay and inter-assay coefficients of variation were 5 and 6.75%, respectively, and the minimum detectable value was 0.3 mg/L. Undetectable samples were assigned half of the minimum detectable value (0.15 mg/L) (Meier-Ewert et al., 2004; Ferrie et al., 2013; Szabo et al., 2020). All samples from the same participant were measured in the same assay.

### Neurobehavioral Performance

A precise computer-based neurobehavioral test battery (NTB) was administered 11 times during the study [Dell Latitude E5420 Laptops; Software: Windows XP; NTB custom reaction time (RT) testing software (Pulsar Informatics, Inc., Philadelphia, PA, United States)]: every day of the 5-day experiment at 1130 and 1730 h, and an additional test at 0400 h after the TSD night (Figure 1). The NTB included the following objective performance measures: the 3-min Psychomotor Vigilance Test (PVT), a behavioral attention test that is practical and applicable to real-world settings (Basner and Rubinstein, 2011; Basner et al., 2011, 2018; Hilditch et al., 2016; Grant et al., 2017; Behrens et al., 2019; Hansen et al., 2019; Benderoth et al., 2021), which measures the total number of lapses (RT > 355 ms) and errors (RT < 100 ms); and the Digit Symbol Substitution Test (DSST) (Hartman, 2009), a cognitive throughput task, which measures the number correct. The KSS (Åkerstedt and Gillberg, 1990) measures self-reported sleepiness. All tests are well-validated measures to examine sleep loss and they show stable, robust individual differences in healthy populations (Dennis et al., 2017; Yamazaki and Goel, 2020; Brieva et al., 2021; Casale et al., 2021b; Yamazaki et al., 2021a), and were administered in the following order during all test bouts: DSST, KSS, 3-min PVT. As such, resilient and vulnerable individuals were determined by a median split on average values from the three NTB sessions during TSD (Patanaik et al., 2015; Moreno-Villanueva et al., 2018; Caldwell et al., 2020) for 3-min PVT total lapses and errors, DSST performance, and KSS scores. We dichotomized participants as such given that, for initial examination and categorization of novel biomarkers, it is more suitable and applicable to create resilient and vulnerable groups in healthy adult samples, as per convention in the field (Chuah et al., 2009; Rocklage et al., 2009; Chee and Tan, 2010; Diekelmann et al., 2010; Patanaik et al., 2015; Yeo et al., 2015; Xu et al., 2016; Moreno-Villanueva et al., 2018; Caldwell et al., 2020; Salfi et al., 2020; Brieva et al., 2021; Casale et al., 2021b; Yamazaki et al., 2021b), especially given our sample size. Importantly, systematic examination of multiple approaches and thresholds for evaluating differential neurobehavioral vulnerability to sleep loss has demonstrated that median splits on averaged performance scores, rather than change from baseline or variance in scores, are consistent indicators of resilience and vulnerability during sleep deprived and well-rested periods (Brieva et al., 2021; Casale et al., 2021b; Yamazaki et al., 2021b), thus further justifying our methods.

### Trier Social Stress Test

The TSST is a well-validated and commonly used test to experimentally induce psychological stress (Allen et al., 2014, 2017). A modified 30-min TSST was conducted with participants remotely *via* audio and a one-way video camera (Moreno-Villanueva et al., 2018). Notably, remote implementation of the TSST is a validated virtual alternative to the traditional in-person method (Kelly et al., 2007; Ruiz et al., 2010; Helminen et al., 2021). The TSST consisted of several challenging interview questions regarding responses to TSD, including those related to motivation, performance, aptitude, and interactions with others, and several difficult cognitive tests, including a 3-min Stroop task and a 5-min calculation task involving counting backward aloud in 13-step sequences (Moreno-Villanueva et al., 2018). The TSST was followed by debriefing following the afternoon biomarker collection.

### Statistical Analyses

All statistical analyses were performed using SPSS v26 (SPSS Inc., IL, United States) with *p* < 0.05 considered statistically significant and all statistical tests were two-tailed. Descriptive statistics characterizing the sample and outcome measures, including the mean, standard deviation (SD), and standard error of the mean (SEM), are indicated in the results, tables, and figures.

A median split on average performance during TSD and psychological stress for each NTB measure defined the NTB resilient and vulnerable groups (Patanaik et al., 2015; Moreno-Villanueva et al., 2018; Caldwell et al., 2020). A median split on pre-study cortisol values and on CRP values defined low and high pre-study cortisol and CRP groups. One-way ANOVAs determined differences between NTB resilient and vulnerable groups as well as, separately, between the high and low cortisol and CRP groups for age (Wener et al., 2000; Yan et al., 2021), body surface area [BSA; a commonly used biometric unit for normalizing physiologic parameters in applied medical settings (Verbraecken et al., 2006)], and actigraphic sleep characteristics across the study. Chi-square tests determined differences between NTB resilient/vulnerable groups and the low/high pre-study cortisol and CRP groups for sex (Allen et al., 2017; Vargas and Lopez-Duran, 2017). Repeated measures (RM) ANOVA tests were conducted with the within-subject factor “time” [biomarkers (cortisol or CRP) across the study at time points: pre-study, baseline, TSD AM, TSD PM, recovery, and post-study], between-subject factor “NTB group” [NTB (3-min PVT, DSST, or KSS) resilient and vulnerable groups], and the interaction “time × NTB group.” *Post hoc* analyses with Bonferroni corrections were used to evaluate significant time effects. Sex, as a between-subject factor, as well as age and BSA, as continuous covariates, were independently added to the statistical model to determine the influence of these factors on the change in cortisol and CRP across the study. RMANOVAs with the within-subject factor “time” [NTB performance/scores (3-min PVT, DSST, or KSS) across the experiment: B2 1730 h, TSD 1730 h, and R1 1730 h], between-subject factor “pre-study group” [pre-study (cortisol or CRP) low or high group], and interaction “time × pre-study group.” *Post hoc* analyses with Bonferroni corrections were used to evaluate significant time effects. Bonferroni-corrected *p*-values are reported for all *post hoc* analyses. Spearman’s relative rank correlations evaluated the relationships between NTB measures, and Pearson correlation coefficients evaluated the relationships between cortisol and CRP across the study.

Studentized residuals beyond ± 3 SD were used to identify outliers for 3-min PVT lapses and errors and KSS scores. Analyses without the outliers were conducted and the results were unchanged; thus, we retained the outliers in the analyses to maximize statistical power. The distribution of cortisol and CRP were skewed according to the Shapiro–Wilk test of normality; thus, we added 0.15 to the undetectable CRP values (to allow for log transformation) and natural log transformed CRP and cortisol data before analysis to improve fit to normal distributions (average *W* = 0.960 and kurtosis = 0.089 for cortisol; average *W* = 0.879 and kurtosis = −0.114 for CRP) (Meier-Ewert et al., 2004; Frey et al., 2007; Minkel et al., 2014; Wright et al., 2015). The Greenhouse–Geisser correction for degrees of freedom was applied for all RMANOVAs to account for sphericity assumption violations indicated by significant Mauchly’s tests for all main analyses [χ^2^(2–14) = 6.44–79.70, *p* = 0.000–0.045], excluding the pre-study low/high cortisol and CRP for KSS RMANOVAs, χ^2^(2) = 0.50–0.59, *p* = 0.746–0.779, as well as cortisol for the sex RMANOVA, χ^2^(14) = 20.755, *p* = 0.109. To account for multiplicity, the false discovery rate correction of Benjamini and Hochberg (1995) [conducted in the R software environment (R Core Team, 2020)] was applied to all *p*-values derived from the sets of RMANOVAs evaluating cortisol and CRP changes across the study and NTB performance/scores across the experiment including covariate analyses. FDR corrected *p*-values are presented. One participant was withdrawn from the study during R1 but returned for post-study data collection. All RMANOVAs and all recovery *post hoc* comparisons did not include this individual’s data (*N* = 31). An error that occurred during blood collection resulted in another participant’s loss of data during post-study. This individual was excluded from all post-study averages of CRP (*N* = 31), as well as from CRP RMANOVAs, in addition to the aforementioned individual who was removed from the study during recovery (*N* = 30). Otherwise, both individuals’ data points were included in analyses to maximize statistical power (*N* = 32).

## Results

### Participant Characteristics

There were no significant differences between NTB resilient and vulnerable groups, *F*(1) = 0.000–2.638, *p* = 0.115–1.000, or between pre-study high and low cortisol or pre-study high and low CRP groups, *F*(1) = 0.000–2.034, *p* = 0.164–1.000, defined by sex, age, or BSA, except for by sex distribution for the 3-min PVT, for which there were significantly more males (males, *N* = 12) in the resilient than vulnerable group, χ^2^(1) = 4.571, *p* = 0.033 (Table 1). However, when 3-min PVT performance during TSD was compared by sex, the group difference was not significant, *F*(1) = 4.031, *p* = 0.054; other NTB measures also did not show significant sex differences in performance, *F*(1) = 0.013–0.993, *p* = 0.327–0.910. During the 5-day experiment (Figure 1), NTB resilient and vulnerable groups did not differ in actigraphic sleep onset latency, wake after sleep onset, or total sleep time, *F*(1) = 0.000–3.755, *p* = 0.062–0.992 (Table 1 shows actigraphic data divided by the 3-min PVT resilient-vulnerable grouping), except that the DSST resilient and the KSS vulnerable groups had significantly shorter onset latencies at B1 than the DSST vulnerable and KSS resilient groups, *F*(1) = 4.380–4.588, *p* = 0.041–0.045, and the DSST vulnerable group had a significantly shorter onset latency at R1 than the DSST resilient group, *F*(1) = 6.491, *p* = 0.016.

**TABLE 1:** Participant characteristics and actigraphic sleep data during the 5-day experiment (mean ± SD).

		All participants	3-min PVT*^A^* resilient	3-min PVT vulnerable
*N*		32	16	16
Sex (female/male)^G^		14/18	4/12	10/6
Age		35.1 ± 7.15	33.1 ± 6.94	37.1 ± 6.99
Body surface area (m^2^)		1.85 ± 0.24	1.90 ± 0.24	1.79 ± 0.23
Baseline 1^B^	TST (min)^D^	405.8 ± 32.4	411.4 ± 37.0	399.8 ± 26.7
	SOL (min)^E^	11.6 ± 17.9	12.9 ± 23.5	10.1 ± 9.34
	WASO (min)^F^	37.1 ± 20.3	30.6 ± 14.2	44.1 ± 23.7
Baseline 2	TST (min)	402.6 ± 35.3	407.2 ± 37.3	398.0 ± 33.7
	SOL (min)	11.5 ± 24.9	15.9 ± 33.8	7.19 ± 9.83
	WASO (min)	38.2 ± 19.7	34.7 ± 14.0	41.8 ± 24.1
Total sleep deprivation	TST (min)	–	–	–
	SOL (min)	–	–	–
	WASO (min)	–	–	–
Recovery 1*^C^*	TST (min)	528.3 ± 69.4	524.3 ± 90.0	532.6 ± 40.2
	SOL (min)	1.81 ± 3.36	2.81 ± 4.17	0.73 ± 1.79
	WASO (min)	51.3 ± 47.3	40.9 ± 38.6	62.3 ± 54.2
Recovery 2*^B^*	TST (min)	390.3 ± 50.1	392.5 ± 42.2	388.2 ± 57.8
	SOL (min)	12.3 ± 13.4	14.0 ± 16.4	10.8 ± 10.0
	WASO (min)	47.3 ± 36.9	39.1 ± 18.3	55.0 ± 47.7

*^A^PVT, Psychomotor Vigilance Test.*

*^B^N = 15 in the 3-min PVT vulnerable group.*

*^C^N = 15 in the 3-min PVT resilient group.*

*^D^TST, total sleep time.*

*^E^SOL, sleep onset latency.*

*^F^WASO, wake after sleep onset.*

*^G^Chi-square test showed a significant difference in sex distribution in the 3-min PVT resilient and vulnerable groups (p = 0.033).*

Cortisol and CRP values were within normal, healthy adult ranges as reported in previous literature (Laudat et al., 1988; Broekhuizen et al., 2006; Hellhammer et al., 2009), except for in two participants. One participant had a pre-study CRP level of 27 mg/L (*z*-score = 4.63); however, for the remaining five time points, this individual’s CRP ranged between 1.22 and 1.37 mg/L. Another participant had CRP levels between 9.17 and 14 mg/L (*z*-scores = 2.17–4.86) throughout the study; however, the results did not change when this participant’s data points were removed. Thus, all data were retained to maximize the number of data points in the analyses. Pearson correlation coefficients between cortisol and CRP were significant at pre-study (*r* = −0.50, *p* = 0.004), recovery (*r* = −0.36, *p* = 0.046), and post-study (*r* = −0.42, *p* = 0.017), but not significant at any other time point (*r* = −0.045 to −0.311, *p* = 0.083–0.809).

### Cortisol

#### Cortisol Profile Between Neurobehavioral Test Battery Resilient and Vulnerable Groups

**FIGURE 2:**
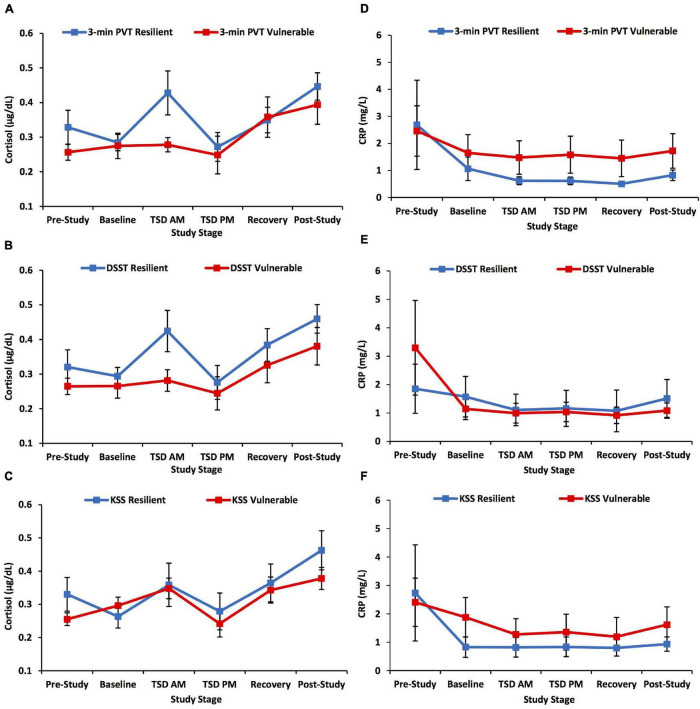
Cortisol and C-reactive protein (CRP) changes across the study defined by a median split on 3-min Psychomotor Vigilance Test (PVT) lapses and errors, Digit Symbol Substitution Test (DSST) total number correct, and Karolinska Sleepiness Scale (KSS) scores. The left panel of graphs shows the pattern of change in cortisol across the study and the right panel of graphs shows the pattern of change in CRP across the study for neurobehavioral measures. Resilient and vulnerable groups were defined by a median split on **(A,D)** 3-min PVT lapses and errors; **(B,E)** DSST total number correct; and **(C,F)** KSS scores. There were no significant findings for the 3-min PVT, DSST, or KSS analyses. *N* = 15 in the recovery resilient points in **(A,B,D,E)** and in the recovery vulnerable points in **(C,F)** due to one participant withdrawn from the study during recovery night 1; *N* = 15 in the post-study resilient points in **(D–F)** due to a blood collection error; all other data points are *N* = 16. Data are not transformed and are presented as mean ± SEM.

The 3-min PVT, DSST, and KSS did not show significant time × group interactions, *F*(3.447–3.551, 99.964–102.972) = 0.479–0.902, *p* = 0.609–0.793, η*_*p*_*^2^ = 0.016–0.030, or significant overall between-subjects effects, *F*(1) = 0.020–2.408, *p* = 0.264–0.889, η*_*p*_*^2^ = 0.001–0.077, for cortisol across the study (Figures 2A–C).

#### Main Effect of Time for Cortisol Across Study

**FIGURE 3:**
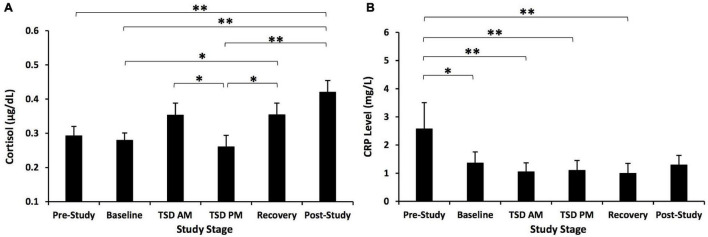
Cortisol and C-reactive protein (CRP) changes across the study. **(A)** Cortisol showed a significant change across the study. *Post hoc* analyses showed that post-study cortisol was significantly higher than pre-study, baseline, and TSD PM cortisol. Cortisol at TSD AM was significantly higher than at TSD PM. Recovery cortisol was also significantly higher than at baseline and TSD PM. **(B)** CRP showed a significant time effect across the study. *Post hoc* analyses showed that pre-study CRP was significantly higher than CRP at all other time points except for post-study. *N* = 31 for the recovery point in **(A)** due to one participant withdrawn from the study during recovery night 1 and for the post-study point in **(B)** due to one participant’s loss of data due to a blood collection error; all other points are *N* = 32. *p < 0.05, **p < 0.01. Data are not transformed and are presented as mean ± SEM.

Cortisol showed a significant time effect across the study, *F*(3.590, 107.714) = 9.563, *p* < 0.001, η*_*p*_*^2^ = 0.242 (Figure 3A). *Post hoc* analyses showed that post-study cortisol was significantly higher than pre-study (*p* = 0.003), baseline (*p* < 0.001), and TSD PM cortisol (*p* = 0.001). Baseline cortisol was significantly lower than recovery cortisol (*p* = 0.025), and TSD PM cortisol was significantly lower than TSD AM cortisol (*p* = 0.020) and recovery cortisol (*p* = 0.025).

We also examined demographic covariates in the statistical model. When sex was added to the model as a between-subject factor, the time × sex interaction was significant, *F*(3.879, 112.505) = 3.429, *p* = 0.036, η*_*p*_*^2^ = 0.106. The time effect was significant in both females, *F*(4.070–52.904) = 12.103, *p* < 0.001, η*_*p*_*^2^ = 0.482, and males, *F*(3.297, 52.747) = 2.769, *p* = 0.046, η*_*p*_*^2^ = 0.148. In females, cortisol was significantly lower at baseline than at post-study, *p* = 0.047, and TSD PM cortisol was significantly lower than at baseline, TSD AM, recovery, and post-study, *p* ≤ 0.001–0.012. In males, cortisol was significantly lower at baseline than at post-study, *p* = 0.002. Females also had significantly lower cortisol than males at TSD PM, *F*(1) = 4.363, *p* = 0.045, η*_*p*_*^2^ = 0.127. There were no significant differences between females and males at any other time point, *F*(1) = 0.044–0.889, *p* = 0.353–0.835, η*_*p*_*^2^ = 0.001–0.030. When age was added to the model, the time × age interaction was not significant, *F*(3.682, 106.767) = 2.142, *p* = 0.219, η*_*p*_*^2^ = 0.069, and the overall time effect was significant, *F*(3.682, 106.767) = 3.889, *p* = 0.022, η*_*p*_*^2^ = 0.118. Lastly, when BSA was added to the model, the time × BSA interaction was not significant, *F*(3.591, 104.147) = 0.922, *p* = 0.607, η*_*p*_*^2^ = 0.031, and the overall time effect was not significant, *F*(3.591, 104.147) = 1.301, *p* = 0.454, η*_*p*_*^2^ = 0.043.

#### Neurobehavioral Test Battery Profiles in High vs. Low Pre-study Cortisol Groups

**FIGURE 4:**
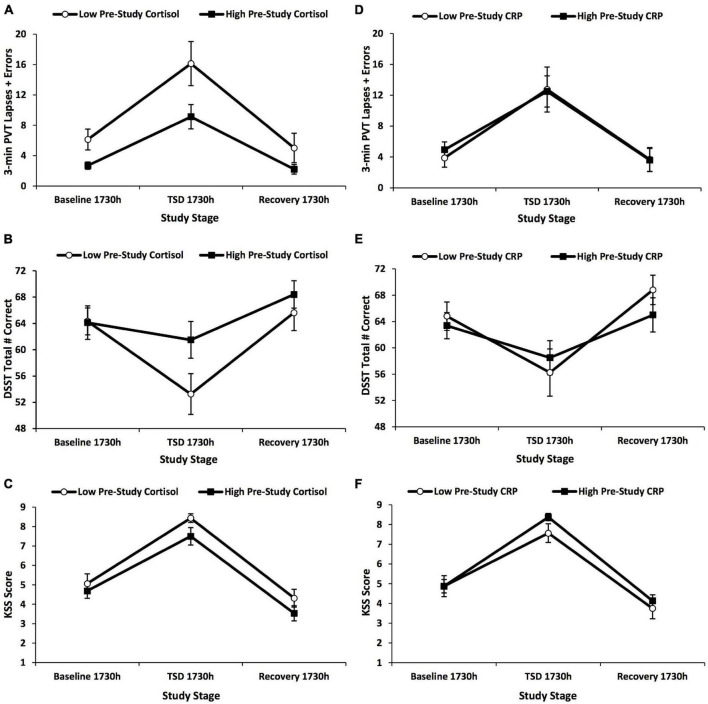
Changes in 3-min Psychomotor Vigilance Test (PVT) lapses and errors, Digit Symbol Substitution Test (DSST) total number correct, and Karolinska Sleepiness Scale (KSS) scores across the 5-day experiment defined by a median split on pre-study cortisol and pre-study C-reactive protein (CRP) values. The graphs show the changes in **(A,D)** 3-min PVT lapses and errors; **(B,E)** DSST total number correct; and **(C,F)** KSS scores by a median split on **(A–C)** pre-study cortisol values and **(D–F)** pre-study CRP values. Three-minute PVT and DSST performance and KSS scores did not show any significant findings for these analyses. *N* = 15 in the recovery points of the high pre-study cortisol group in **(A–F)** due to one participant withdrawn from the study during recovery night 1; all other data points are *N* = 16. Data are mean ± SEM.

The high vs. low pre-study cortisol groups did not show a significant time × group interaction, *F*(1.612–1.959, 46.759–56.824) = 0.418–3.630, *p* = 0.116–0.767, η*_*p*_*^2^ = 0.014–0.111, or significant between-subject effects, *F*(1) = 1.407–4.455, *p* = 0.116–0.396, η*_*p*_*^2^ = 0.046–0.133, for 3-min PVT and DSST performance, or KSS scores across the study (Figures 4A–C).

### C-Reactive Protein

#### C-Reactive Protein Profile Between Neurobehavioral Test Battery Resilient and Vulnerable Groups

The 3-min PVT, DSST, and KSS did not show significant time × group interactions, *F*(2.939–2.998, 82.303–83.944) = 0.135–1.264, *p* = 0.681–0.938, η*_*p*_*^2^ = 0.005–0.043, or significant overall between-subject effects, *F*(1) = 0.350–1.262, *p* = 0.670–0.812, η*_*p*_*^2^ = 0.012–0.043, for CRP across the study (Figures 2D–F).

#### Main Effect of Time for C-Reactive Protein Across the Study and With Covariates

C-reactive protein also showed a significant time effect across the study, *F*(3.007, 87.213) = 10.09, *p* < 0.001, η*_*p*_*^2^ = 0.258 (Figure 3B). *Post hoc* analyses showed that pre-study CRP was significantly higher than baseline, TSD AM, TSD PM, and recovery CRP (*p* = 0.001–0.014).

We also examined demographic covariates in the statistical model. When sex was added to the model as a between-subject factor, the time × sex interaction was not significant, *F*(3.033, 84.936) = 2.692, *p* = 0.162, η*_*p*_*^2^ = 0.088, and the between-subject effect was not significant, *F*(1) = 3.367, *p* = 0.218, η*_*p*_*^2^ = 0.107. When age was added to the model as a covariate, the time × age interaction was not significant, *F*(3.014, 84.398) = 1.262, *p* = 0.650, η*_*p*_*^2^ = 0.043, and the overall time effect was not significant, *F*(3.014, 84.398) = 0.448, *p* = 0.910, η*_*p*_*^2^ = 0.016. Lastly, when BSA was added to the model as a covariate, the time × BSA interaction was not significant, *F*(2.967, 83.071) = 0.334, *p* = 0.910, η*_*p*_*^2^ = 0.012, and the overall time effect was not significant, *F*(2.967, 83.071) = 0.328, *p* = 0.910, η*_*p*_*^2^ = 0.012.

#### Neurobehavioral Test Battery Profiles in High vs. Low Pre-study C-Reactive Protein Groups

The high vs. low pre-study CRP groups did not show a significant time × group interaction, *F*(1.580–1.965, 45.827–56.994) = 0.110–1.675, *p* = 0.566–0.908, η*_*p*_*^2^ = 0.004–0.055, or between-subject difference, *F*(1) = 0.030–0.572, *p* = 0.812–0.908, η*_*p*_*^2^ = 0.001–0.019, for 3-min PVT and DSST performance, or KSS scores across the study (Figures 4D–F).

### Neurobehavioral Deficits Induced by Sleep Loss and Psychological Stress

Three-minute PVT performance, DSST performance, and KSS scores were all significantly, negatively affected by TSD and psychological stress, *F*(1.580–1.965, 45.827–56.994) = 17.878–90.551, all *p*’s < 0.001, η*_*p*_*^2^ = 0.381–0.757. Table 2 presents the mean and SD for participants on each NTB measure during averaged study periods [baseline, TSD, recovery (averaged from R1 and R2)] and by NTB resilient and vulnerable groups. Performance and self-report scores all returned to baseline levels (all *p*’s ≤ 0.001) except for the KSS, in which recovery sleepiness scores were significantly lower than at baseline (*p* = 0.013).

**TABLE 2:** Neurobehavioral measures during baseline, total sleep deprivation (TSD), and recovery*^A^* during the 5-day experiment (mean ± SD).

Measure	Baseline	TSD	Recovery
3-min PVT^B^ lapses and errors	3.53 ± 4.07	11.0 ± 7.96	3.62 ± 3.94
3-min PVT resilient group	1.59 ± 1.63	5.19 ± 2.79	1.73 ± 1.60
3-min PVT vulnerable group	5.63 ± 4.72	16.69 ± 7.00	5.39 ± 4.67
DSST^C^ total # correct	63.68 ± 8.44	58.39 ± 9.49	67.85 ± 9.12
DSST resilient group	68.56 ± 7.51	65.71 ± 5.18	73.92 ± 7.63
DSST vulnerable group	58.47 ± 5.77	51.17 ± 6.37	62.17 ± 6.39
KSS^D^ score	4.48 ± 1.68	7.73 ± 1.02	3.87 ± 1.47
KSS resilient group	3.72 ± 1.61	7.02 ± 0.91	3.11 ± 1.00
KSS vulnerable group	5.34 ± 1.33	8.52 ± 0.40	4.68 ± 1.47

*^A^Measure scores were averaged by study stage [two neurobehavioral test batteries (NTBs) for baseline, three NTBs for TSD, four NTBs for recovery (from recovery night 1 and recovery night 2)].*

*^B^PVT, Psychomotor Vigilance Test.*

*^C^DSST, Digit Symbol Substitution Test.*

*^D^KSS, Karolinska Sleepiness Scale.*

During sleep deprivation and psychological stress, the range of the Spearman relative rank correlations between the two objective performance measures (3-min PVT and DSST) was ρ = −0.579, *p* = 0.001, and the range between the objective performance measures and the KSS was ρ = −0.142–0.371, *p* = 0.036–0.439. The profiles of change in NTB performance and self-report scores with sleep loss and psychological stress are comparable to results obtained in laboratory studies (Dennis et al., 2017; Yamazaki and Goel, 2020; Brieva et al., 2021; Casale et al., 2021b; Yamazaki et al., 2021a,b).

## Discussion

Cortisol and CRP levels significantly changed across our study, which included two commonly experienced stressors, TSD and psychological stress. Cortisol, but not CRP, increased after a night of TSD. Furthermore, during recovery, cortisol levels did not return to pre-TSD levels, but CRP levels did not differ from those during baseline. As expected, cognitive performance and self-reported sleepiness worsened with TSD and stress. However, cortisol and CRP did not show significant differences between resilient vs. vulnerable groups, and pre-study low vs. high cortisol/CRP groups did not significantly differ in performance or sleepiness. Thus, using our design, both biological markers are not reliable discriminators of cognitive performance or self-reported sleepiness during TSD and psychological stress in our healthy population.

Cortisol increased after a night of TSD in all participants, consistent with some prior studies using healthy samples (Leproult et al., 1997; Wright et al., 2015; Choshen-Hillel et al., 2021; Lamon et al., 2021), but not with others (Åkerstedt et al., 1980; Vgontzas et al., 2004; Frey et al., 2007; van Leeuwen et al., 2009; Pejovic et al., 2013; Honma et al., 2020). Cortisol also decreased from TSD AM to TSD PM, reflecting its well-established, time-of-day profile (Krieger et al., 1971; Czeisler et al., 1999). However, one prior study in a healthy sample (Vargas and Lopez-Duran, 2017) found a blunted cortisol response to the TSST when it was administered in the late morning/early afternoon (similar timing to that in our protocol) during acute TSD. These results are consistent with our findings, since the TSST in our study occurred shortly before the TSD PM biomarker collection, and in which we detected a decrease in cortisol. Nevertheless, due to our study design, we are not able to parse the time-of-day effect of cortisol from a blunted cortisol response. Additionally, we found a significant time × sex effect, whereby females showed lower cortisol at TSD PM compared to all other time points except at pre-study, while in males the TSD PM cortisol did not differ from any other study time point. This finding is interesting in the context of prior literature suggesting that sex differences exist in cortisol responses to sleep loss (Vargas and Lopez-Duran, 2017) and stress (Allen et al., 2017). Our results underscore the importance of considering such demographic variables as covariates when evaluating cortisol under sleep loss and stress and warrant further investigation.

During recovery, cortisol was greater than both at pre-study and baseline in our overall sample. Only a few studies have reported how cortisol levels after recovery from sleep deprivation compare to levels during baseline before sleep deprivation. Notably, our results contrast with a prior study comparing baseline cortisol to recovery cortisol after 48 h of TSD (Åkerstedt et al., 1980), and with studies comparing baseline cortisol to recovery cortisol after SR or acute TSD, which showed no significant difference (van Leeuwen et al., 2009; Honma et al., 2020) or a decrease (Pejovic et al., 2013) in cortisol between baseline and recovery. The discrepancies between our results and those of other studies may be due to the severity of the sleep loss condition, differences in the time elapsed between the end of the sleep loss and recovery cortisol sample acquisition, or the addition of the TSST stressor, which was not implemented in prior studies (Åkerstedt et al., 1980; van Leeuwen et al., 2009; Pejovic et al., 2013; Honma et al., 2020). The combination of sleep loss and the TSST was an important component of our study since it simulated stressors commonly experienced in applied settings (Barger et al., 2014; Cromwell et al., 2021). Of note, the timeline of neurobehavioral recovery for some measures differs after SR compared to TSD (Yamazaki et al., 2021a); thus, perhaps the timeline of physiological recovery after SR vs. TSD also varies. More research investigating recovery profiles of cortisol after sleep loss is warranted.

We found a significant change in CRP throughout our study. The significant decrease in CRP from pre-study to both TSD AM and TSD PM is consistent with one previous study (Frey et al., 2007), but differs from other studies that showed increases (Meier-Ewert et al., 2004; van Leeuwen et al., 2009) or no changes in CRP (Faraut et al., 2011; Irwin et al., 2016; Choshen-Hillel et al., 2021) during sleep loss. We also found that recovery levels were similar to those at baseline, in contrast to another study that found recovery CRP after TSD was significantly higher than baseline CRP (Meier-Ewert et al., 2004). Two studies compared recovery CRP after SR to baseline CRP and found no difference (Faraut et al., 2011) or an increase (van Leeuwen et al., 2009) in CRP from baseline to recovery. Similar to cortisol, possible explanations for the discrepancies in changes in CRP in the present study and in the prior literature include differences in sleep loss protocol/severity of sleep loss (hours of sleep deprivation, TSD vs. SR), time elapsed between the end of sleep loss and recovery CRP sample acquisition, or the addition of the TSST in our study. However, it is notable that in our study, CRP levels did return to baseline levels, thus suggesting that two nights of recovery sleep following TSD may mitigate the sleep-loss related decrements. Additionally, when age and BSA were independently added to the analysis model as covariates, the time effect was no longer significant. Given the literature on the relationship between age and CRP (Wener et al., 2000; Yan et al., 2021), as well as body weight and CRP (Choi et al., 2013; Cohen et al., 2021), future research should investigate the influence of age and BSA on CRP across sleep loss and stress using larger sample sizes.

Interestingly, there were significant negative correlations between cortisol and CRP at pre-study, recovery, and post-study, but not at baseline, TSD AM, or TSD PM. This suggests that although cortisol and CRP tap into different biological dimensions as part of discrete neurobiological systems (cortisol is an HPA axis marker and CRP is an inflammatory marker), the two metabolites may be related at certain non-sleep loss/stress time points. Indeed, while cortisol demonstrates diurnal variability and rapidly changes in response to stressors, CRP does not appear to vary by time of day in our results and in those from a different study (Meier-Ewert et al., 2001), nor does it seem to change rapidly in response to stressors.

For the first time, we investigated whether cortisol and CRP could discriminate cognitive performance and subjective sleepiness resilience during the combination of TSD and psychological stress. Our results indicate that cortisol and CRP are not markers of neurobehavioral resilience to TSD and stress, at least using our study design. Our findings have important implications for applied settings, including space flight (Barger et al., 2014; Cromwell et al., 2021). We conducted our study in NASA’s HERA missions, which is useful for examining the behavioral health impacts of various stressors, such as sleep loss and isolation experienced during spaceflight (Barger et al., 2014; Cromwell et al., 2021); our results demonstrate the criticality of considering differential vulnerability to sleep loss and stress of astronauts enduring short and long duration missions. Additionally, they have potential implications and applications beyond sleep deprivation and psychological stress. Higher cortisol is associated with obsessive compulsive disorder (Sousa-Lima et al., 2019), depression (Nandam et al., 2020), cardiovascular risk factors (Kelly et al., 1998; Iob and Steptoe, 2019), and declining cognitive performance in older individuals (Ouanes and Popp, 2019). The increase in cortisol that we observed in the entire sample during TSD AM and the sustained increased levels during recovery, may indicate that repeated exposure to TSD could cause repeated increased and sustained cortisol levels, which may lead to greater risk of the aforementioned adverse health outcomes, though further research is needed in studies involving a control group.

Further research is also needed concerning the underlying neural correlates of differential resilience and vulnerability to sleep deprivation in relation to various neurobehavioral metrics and biomarkers. While prior studies have reported which brain regions are primarily recruited by specific neurobehavioral tasks and how these associations are impacted by sleep deprivation – the PVT recruits regions responsible for vigilant attention (i.e., the prefrontal cortex, the motor cortex, the inferior parietal cortex, and the visual cortex) (Nasrini et al., 2020; Smith et al., 2021), the DSST recruits regions associated with complex scanning and visual tracking (i.e., the temporal cortex, the prefrontal cortex, and the motor cortex) (Nasrini et al., 2020; Smith et al., 2021), and the KSS recruits regions related to attention and sensory transmission (i.e., the thalamus and the right middle frontal gyrus) (Sun et al., 2020; Motomura et al., 2021) – more work is still needed in this area. Particularly, identifying neural signatures of neurobehavioral resilience and vulnerability to sleep deprivation *via* neural imaging techniques would provide additional methods for determining such individual differences. To our knowledge, very few studies have investigated this concept (Chee and Tan, 2010; Li et al., 2020; Zhu et al., 2020), thus warranting the need for future work.

There are a few limitations to this study. All participants were healthy adults; thus, our data may not be generalizable to clinical populations, such as individuals with PTSD, since these individuals generally have lower basal cortisol (Pan et al., 2018). Similarly, our participants were young to middle aged adults. Adolescents and older individuals may show different changes in response to sleep deprivation and psychological stress, although this contrasts one finding showing cortisol responses during the TSST while sleep deprived did not differ in older adults (60–72 years old) compared to healthy, younger adults (18–30 years old) (Schwarz et al., 2018). Thus, future studies should investigate the possibility that other populations may respond differently to sleep loss and psychological stress. Additionally, the fasting period before biomarker collection may have slightly impacted cortisol and CRP levels, though previous studies have shown no significant effect of fasting on baseline levels of these biomarkers (Kirschbaum et al., 1997; Dumanovic et al., 2021). Similarly, neurobehavioral test bouts were not performed during the fasted state (other than the TSD 0400 h test bout), so fasting also likely had no influence on performance metrics. Due to protocol restrictions, we were unable to collect cortisol samples immediately following exposure to the TSST and therefore, we could not capture peak cortisol occurring in response to the TSST. We also could not systematically assess potential effects of psychological stress and/or TSD on circadian phase, since biomarkers were only collected at one time point each day (except on the afternoon of TSD). In addition, the lack of a control group consisting of the TSST without TSD limits differentiation of the impact of sleep loss separately from that of stress in this study. However, it is plausible that in high-pressure applied settings, such as space flight, individuals will likely experience both sleep loss and stress (Barger et al., 2014; Cromwell et al., 2021), thus retaining the usefulness of our results, despite the lack of a control group. Our results are also limited in generalizability to the tests administered and administration environment, since use of other neurobehavioral measures or testing in real-world situations may yield different results.

In conclusion, both cortisol and CRP levels significantly changed across our study, with cortisol, but not CRP, increasing with TSD. In addition, cortisol levels did not return to pre-TSD levels during recovery, but CRP levels did not differ from those during baseline. Although cognitive performance and self-reported sleepiness worsened with TSD and stress, cortisol and CRP did not show significant differences between resilient vs. vulnerable groups, and pre-study low vs. high cortisol/CRP groups did not significantly differ in performance or sleepiness. Thus, cortisol and CRP are not reliable discriminators of neurobehavioral performance during TSD and psychological stress in our healthy population using our study design.

## Data Availability Statement

The data generated and analyzed during the current study are available from the corresponding author upon reasonable request.

## Ethics Statement

The studies involving human participants were reviewed and approved by the Institutional Review Boards of NASA and of the University of Pennsylvania. The participants provided their written informed consent to participate in this study.

## Author Contributions

NG designed the overall study and provided the financial support. EY and CA conducted statistical analyses of the data. EY, CA, CC, LM, AE, and NG prepared the manuscript. All authors reviewed and approved the final manuscript.

## Conflict of Interest

The authors declare that the research was conducted in the absence of any commercial or financial relationships that could be construed as a potential conflict of interest.

## Publisher’s Note

All claims expressed in this article are solely those of the authors and do not necessarily represent those of their affiliated organizations, or those of the publisher, the editors and the reviewers. Any product that may be evaluated in this article, or claim that may be made by its manufacturer, is not guaranteed or endorsed by the publisher.
